# Audit Review of GP Referrals to Perinatal Mental Health Team – Emphasis on Medication

**DOI:** 10.1192/bjo.2024.513

**Published:** 2024-08-01

**Authors:** Shirley Roy, Paul Chima

**Affiliations:** PMHT, Coventry, United Kingdom

## Abstract

**Aims:**

Audit had been completed with aim to review GP referrals to Perinatal Mental Health Services over a 6/12 month period.

Focus on medication, and information provided on referral proforma; prescribing via letters sent to Perinatal Mental Health Services.

The reason for undertaking this project is due to evidence of variance in practice in prescribing and documenting medications.

**Methods:**

The project team retrospectively took 6 months of data each for the four localities and looked at the list from the weekly MDT during that period.

The team identified the GP referrals and then looked in detail at the referral in Carenotes System.

The data was collected on a proforma designed in Microsoft Word and was then sent to the Improvement Team for collation and analysis using Microsoft Excel.

**Results:**

66% used the referral proforma and 20% used the referral letter.

The majority (106) of referrals were for a routine review/nonspecific.

The majority (78) of referrals were post-natal. 25% of referrals did not indicate whether the patient was post-natal or antenatal and hence no Expected Date of Delivery [MS(CPT1] entered.

10% of referrals medication had been stopped. 24% of patients were to review to start medication.

Results show that sertraline had been initiated the most frequently. 65% unspecified. In 26%, sertraline had been most frequently prescribed.

Where medication had been stopped, the majority of proformas (64%) were incomplete. 9% of patients had Selective Serotonin Reuptake Inhibitors suspended such as sertraline and citalopram.

**Conclusion:**

In most cases, the reason for referral was unclear.

Medication was often stopped unnecessarily – for most medications, it was not indicated whether medication was started/stopped.

If patients were started on medication, sertraline and citalopram were either started or stopped most frequently.

We also found that some of the referrals were illegible.

We presented the findings within our perinatal mental health team meeting.

We found the following to be actioned, including discussions with local GP practices and/or local GP educational forums.

We hope to re audit following the above action.

